# The Role of Calender Gap in Barrel and Screw Wear in Counterrotating Twin Screw Extruders

**DOI:** 10.3390/polym13070990

**Published:** 2021-03-24

**Authors:** Abdullah Demirci, Ismail Teke, Nickolas D. Polychronopoulos, John Vlachopoulos

**Affiliations:** 1 Department of Mechanical Engineering, Yildiz Technical University, 34349 Istanbul, Turkey; iteke@yildiz.edu.tr; 2 Mikrosan Inc., 41400 Gebze, Turkey; 3 Polydynamics Inc., Dundas, ON L9H 6Y3, Canada; polyrheo@polydynamics.com; 4 Department of Chemical Engineering, McMaster University, Hamilton, ON L8S 4L7, Canada; vlachopj@mcmaster.ca

**Keywords:** closely intermeshing, separating forces, screw deflection

## Abstract

It has been known in the industrial sector that in closely intermeshing counterrotating twin screw extruders, large separating forces develop in the calender gap, which push the screws towards the barrel wall. The result is significant wear in the region defined by 30°- and 60°-degree angles from the vertical. In the present investigation, pressures were measured around the barrel in extrusion of two rigid PVC resins in a laboratory extruder of 55 mm diameter and the forces on the screw core were determined. Numerical flow simulations were also carried out using the power-law viscosity parameters of the resins. From the experimental results, it was determined that the resultant forces are in the 30 degree angle direction, and from the computer simulations, the angle is between 18° and 25°. It is argued that the resultant force angle will be somewhat larger in large diameter extruders, due to the additional contribution of gravity.

## 1. Introduction

**Figure 1:**
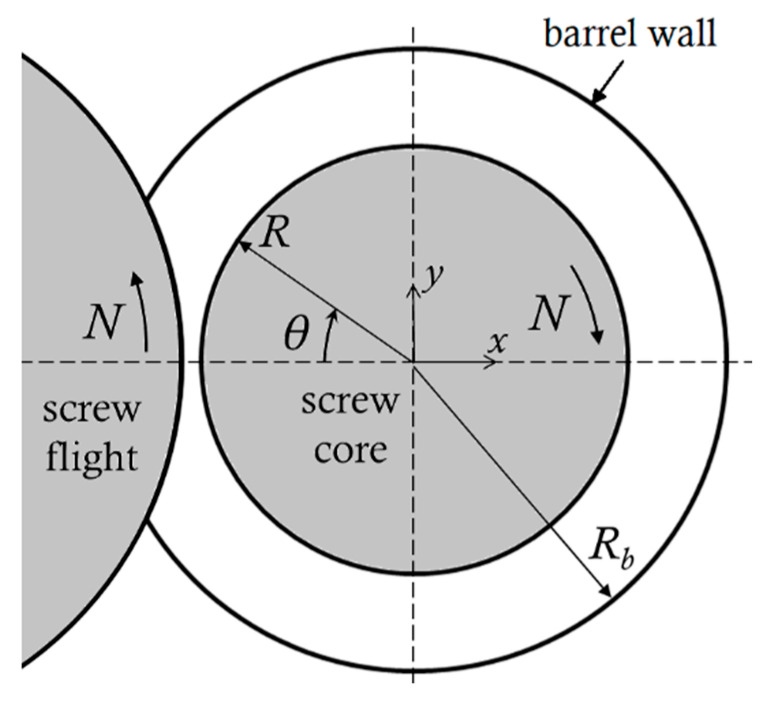
Calender gap between a screw core and screw flight.

The counter-rotating intermeshing twin screw extruders are used widely for pipes, profiles and sheet extrusions. The main advantage over single screw extruders and co-rotating twin screw extruders is their positive displacement pumping, which imparts little frictional heating to temperature sensitive materials, such as PVC. Of course, positive displacement implies limited mixing. The screws form helically distorted C-shaped chambers, as explained in various books [1,2,3,4,5,6,7], which enclose and transport the material axially. The flights of one screw penetrate the channels of the other and the screw velocities are in the same direction in the intermeshing region formed by the core of one screw and the tip of the flight of the other (Figure 1). As the material is dragged in the nip region pressure and separating forces develop, akin to those in the process of calendering [8,9,10,11,12]. The separating force increases as the speed increases and the gap decreases. In closely intermeshing counter-rotating twin screw extruders, used in extrusion of pipes, profiles and sheet, the gaps are rather small and the separating forces very large, which can cause screw deflection. Such extruders run at low rotational speeds to avoid the generation of large separating forces.

Historically, counter-rotating twin screw extruders have received significantly less research and development efforts than corotating. According to Thiele [13] “Dr. White is considered by many to be the historian of the industry. It can be deduced from his book, *Twin-Screw Extruders*, that circumstances and politics played at least some role in classical co-rotators receiving 20 to 50 times greater investments of time and money than classical counter-rotation”. Thiele goes on to acknowledge the limitations in mixing in counterrotating twin screw extruders (TSEs) and to state that: “At high screw speeds the calender pressures became so great as to cause the melt film against barrels opposite the intermesh to fail; that is, the extruder could eat itself. For that reason, production classical counter-rotators were generally limited to 150 RPM and below”. In fact, large diameter closely intermeshing counter-rotating TSEs are limited to less than 50 RPM, according to Martin [6]. For larger gap the separating force would decrease, and such a configuration would allow higher rotational speeds. However, the net positive displacement pumping capacity, provided by close intermeshing, would be reduced or eliminated.

**Figure 2:**
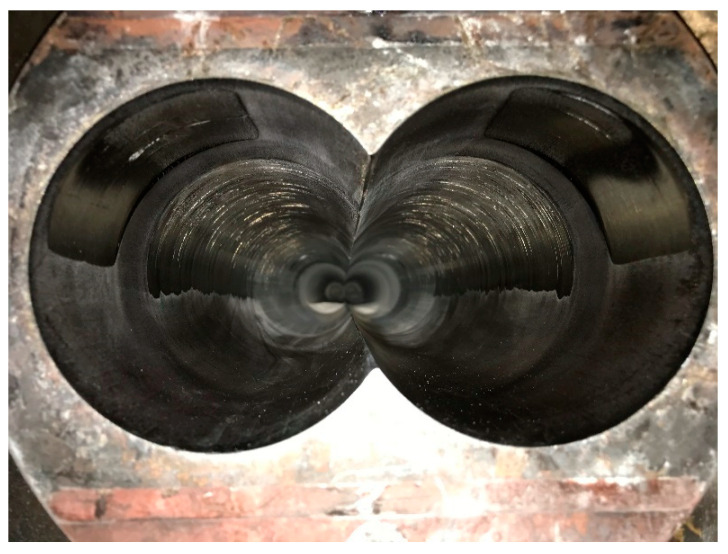
Wear in a nitrided parallel twin barrel, 90 mm screw diameter, 26 L/D ratio. It was run with a highly filled PVC formulation for more than 10,000 h. Highest wear seems to be between the 30° and 60° angle from the vertical.

Intermeshing counter-rotating screws usually rotate outward. It is known in the industrial sector and it has been reported in some books and publications [7,14,15,16,17] that the separating forces are large enough to bend the screws in such a way as to cause maximum wear at the 10 o’clock–2 o’clock position. Although it is known that the forces are large, can bend the screws and cause severe screw and barrel wear, as shown in Figure 2, there have not been any quantitative studies available in the open literature. With the objective of elucidating the pressures and forces developed as a result of the squeeze flow in the calender gap, an experimental and numerical flow simulation study was carried out.

## 2. Equipment and Rheological Characterization 

### 2.1. Equipment

**Figure 3:**
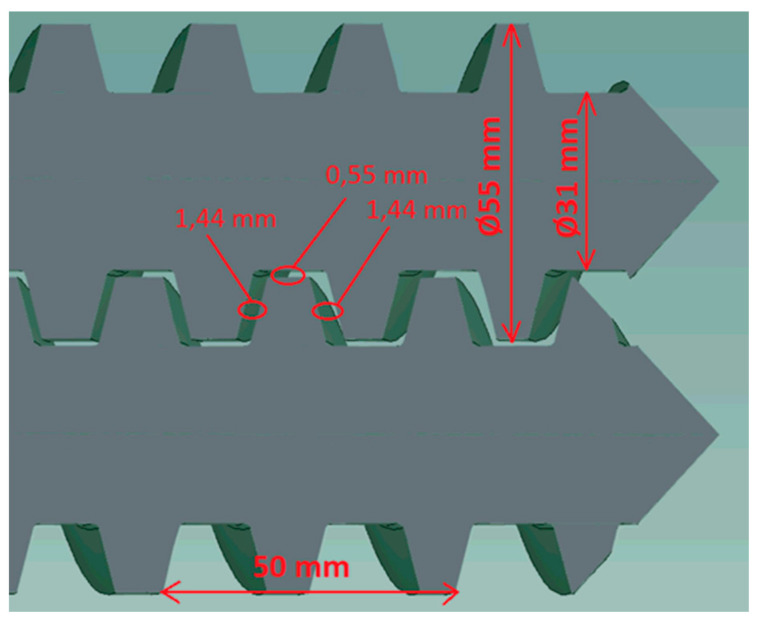
Screw metering zone geometry.

**Figure 4:**
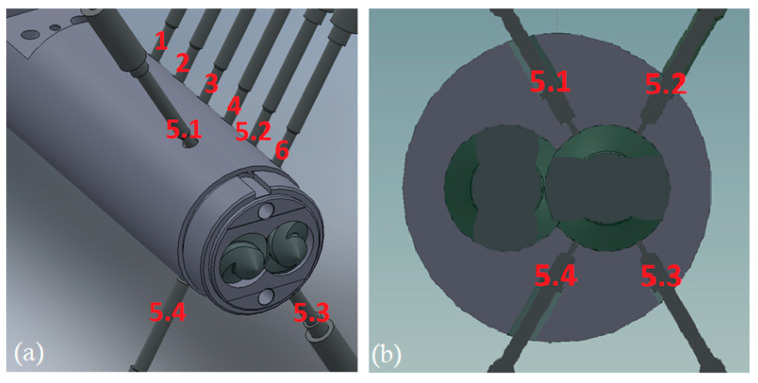
(**a**) Computer generated sketch of the metering zone of the TSE and (**b**) location of pressure sensors at position P5.

A parallel closely intermeshing counter-rotating twin screw extruder of 55 mm diameter and L/D ratio of 20 was used in the experiments. The metering zone has a length of 300 mm and double flight screw geometry with screw pitch of 50 mm, core diameter of 31 mm, screw-barrel gap 0.1 mm, calender gap 0.55 mm and screw flank gap 1.44 mm, as shown in Figure 3. Pressure was recorded by sensors along the barrel, placed at equal distances apart, and at position 5, sensors were located around the barrel at 60°-, 120°-, 240°- and 300°-degree angles, as shown in Figure 4. The extruder was also equipped with a die having an adjustable valve, which could generate pressures up to 70 MPa.

### 2.2. Rheological Characterization of Materials

Two different uPVC powder grade formulations (Table 1), provided by Mikrosan Inc. (Gebze, Turkey), were used in the experiments. The stabilizer package also includes lubricants. These PVC formulations are commercial grades used for cable duct (resin A) and window profile (resin B) extrusion applications [18]. The formulations were mixed in a Zeppelin Reimelt Henschel FML10/KM 23 hot-cold mixer (Kassel, Germany). A Ceast (Pianezza, Italy) Smart Rheo 2000 model capillary rheometer was used for viscosity measurements. The viscosity measurements, were fitted to a power-law model:

(1) $$\eta =m{\dot{\gamma }}^{n-1}$$

where for resin A, *m* = 89,120 Pa∙s^n^ and *n* = 0.434 at 190 °C, and for resin B, *m* = 149,818 Pa∙s^n^ and *n* = 0.4 at 190 °C. 

## 3. Pressure Measurements and Analysis

**Figure 5:**
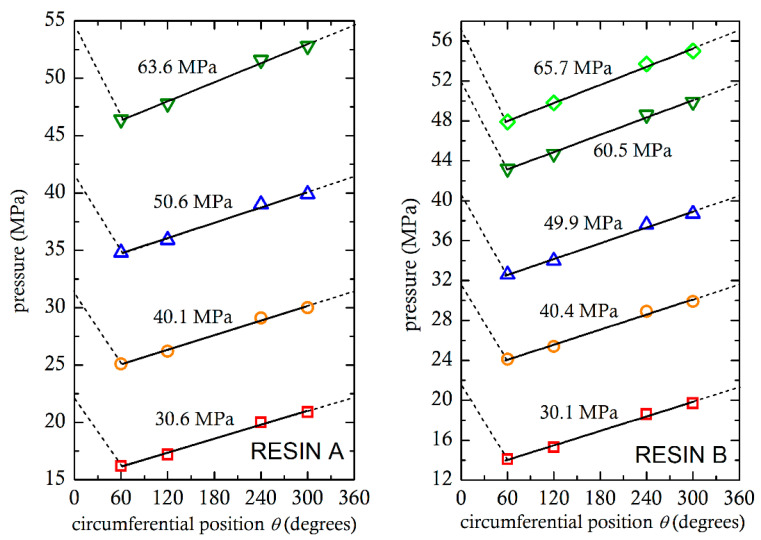
Average pressures measured (for different back pressures). Broken lines are extrapolations to 360°.

By using the adjustable valve in the die, it was possible to generate high pressures in the metering zone and determine to what extent the extruder was filled with molten material. This could be observed from the pressures recorded along the barrel P1, P2, P3, P4, P5 and P6. At location 5, pressures were measured around the barrel at the positions P5.1, P5.2, P5.3 and P5.4, as shown in Figure 5, which is the area of interest in this investigation. As expected, the pressures recorded were fluctuating due to the passage of the screw flights in front of the sensors. However, the average measured pressures shown in Figure 5 were reproducible. The temperatures in the adapter were fairly stable, ranging for 193 °C to 196 °C in the four experiments for resin A and from 194 °C to 197 °C in those for resin B.

It can be seen that the pressures measured increase, from the 60° to the 300° position as the molten PVC is dragged to the nip region. The differences between the maximum (at 300°) and minimum (at 60°) recorded pressures vary from 4.7 MPa to 6.4 MPa for resin A and 5.8 MPa to 7.1 MPa for resin B. The differences are somewhat higher at higher back (adapter) pressures, because the viscosity of polymers increases with pressure [7,11]. From the analyses of the calendering process [8,9,10,11,12], it is well known that the maximum pressure occurs just before the minimum gap. From Middleman [8], the maximum calender pressure is given by:

(2) $$P=m{\left(\frac{U}{H_{o}}\right)}^{n}{\left(\frac{2R}{H_{o}}\right)}^{1/2}\mathbb{P}\left(n\right)$$

where *m* is the consistency index, *n* the power-law index, U the rotational speed, *H_o_* the minimum gap, *R* the radius of roll and ℙ(*n*) is a function of *n* equal to 0.75 for *n* = 0.434 and equal to 0.78 for *n* = 0.4.

From Equation (2), assuming 2*R* = (55 + 31)/2 = 43 mm, for resin A we obtain *P*_max_ = 3.96 MPa and for resin B *P*_max_ = 5.99 MPa. These values are close to the pressure differences measured, despite the fact that Equation (2) was derived for two equal diameter rolls, from an analysis of flow in the vicinity of the minimum gap, and there is no barrel wall involved in the calendering process. Consequently, a rough estimate of the separating force can also be obtained, from Middleman [8]:

(3) $$\frac{F}{L}=m{\left(\frac{U}{H_{o}}\right)}^{n}RF\left(n\right)$$

where *L* is the length of the roll (core of the screw) and F(*n*) = 0.3 for *n* = 0.434 and *F*(*n*) = 3.2 for *n* = 0.4. This equation gives *F*/*L* = 38,104 N/meter for resin A and *F*/*L* = 59784 N/meter for resin B. Assuming that the force is exerted on the entire length of the screw in the 30 cm metering zone, we have for resin A, *F* = 11,431 N (1.16 ton-force) and for resin B, *F* = 17,935 (1.83 ton-force). Obviously, under the action of a separating force of over one metric ton, the screws will be pushed towards the barrel wall and exert abrasive action, as explained also in the introduction of this paper.

**Figure 6:**
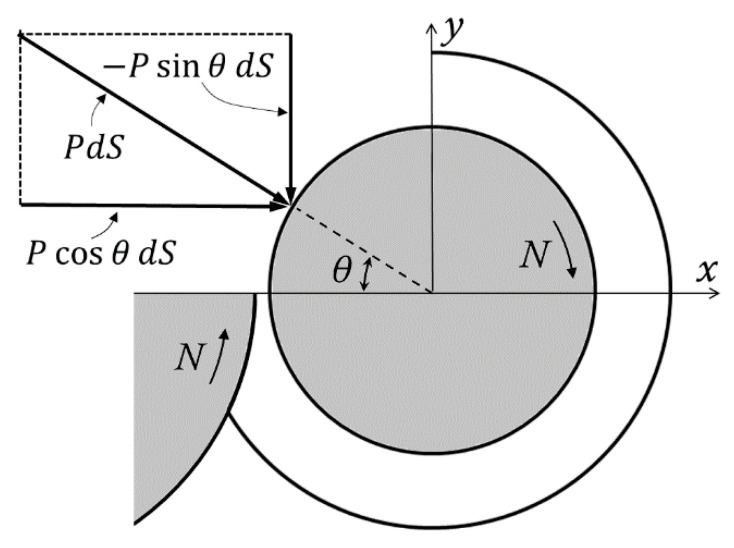
Force components on the screw core.

Another calculation of the separating forces both in the *x* and the *y* directions can be made from the measured pressures at positions P5.1, P5.2, P5.3 and P5.4. These pressures were plotted in Figure 5. The maximum pressure would be just upstream of the nip, which is impossible to measure. However, it can be seen from Figure 5 that measured pressures can be fit to straight lines. It is reasonable to assume that the straight lines can be extended to the nip (360°). It is further assumed that there are no pressure differences in the radial direction. Pressure is normal to the screw core surface and the two components of the force per unit length in the x and y directions of Figure 6 on an infinitesimal surface d*S* = *R*d*θ* will be *p*(*θ*)cos*θ*d*S* = *p*(*θ*)cos*θR*d*θ* and −*p*(*θ*)sin*θ*d*S* = −*p*(*θ*)cos*θR*d*θ*, respectively. The forces per unit length can be obtained by numerically integrating the pressures shown in Figure 5 from 0 to 2π, using the following equations:

(4) $$F_{x}=\int p\left(\theta \right)\cos\theta Rd\theta$$

(5) $$F_{y}=-\int p\left(\theta \right)\sin\theta Rd\theta$$

These two equations are used routinely for load determination in the lubrication of journal bearings [19,20,21]. The results of the integrations are shown in Table 2 and Table 3. The resultant force is at an angle of about 30° with the +*y* axis.

## 4. Numerical Flow Simulations

Various aspects of flow analysis and computer simulations of counter-rotating TSEs [22,23,24,25,26,27,28,29,30,31] have provided significant insights into the flow phenomena. There have not been any publications on the development of the separating forces in the calender gap, which is the objective of this paper. In this investigation, we use the open-source software OpenFOAM [32]. It is computational fluid dynamics software, based on the finite volume method, which we have tested and successfully used for several other problems involving Newtonian, shear-thinning and viscoelastic fluid models [33,34]. We assume that the polymer melt is an incompressible fluid; the flow is creeping (i.e., *Re* << 1) and isothermal. Under these approximations the Navier-Stokes equations are simplified to:

(6) 0=−∇p+∇·τ

where *p* is the pressure and **τ** is the stress tensor. The Generalized Newtonian fluid is used to relate the fluid stresses with the rate of strain given by:

(7) $$\tau =\eta \left(2D\right)$$

where *η* is the non-Newtonian viscosity of the material and **D** is the rate of strain tensor given by:

(8) $$D=\frac{1}{2}\left[\nabla U+\nabla U^{T}\right]$$

where **U** is the velocity vector. We further assume that the fluid rheology is described by the power-law model:

(9) $$\eta =m{\left(II_{D}\right)}^{\frac{n-1}{2}}$$

where *m* is usually referred to as the consistency index (Pa·s^n^), *n* is the power-law index (for *n* = 1 Newtonian fluid) and *II*_D_ is the second invariant [7] of the rate of strain tensor **D**. It can easily be shown that this generalization reduces to the power-law fluid model for simple shear flow, i.e., Equation (1).

**Figure 7:**
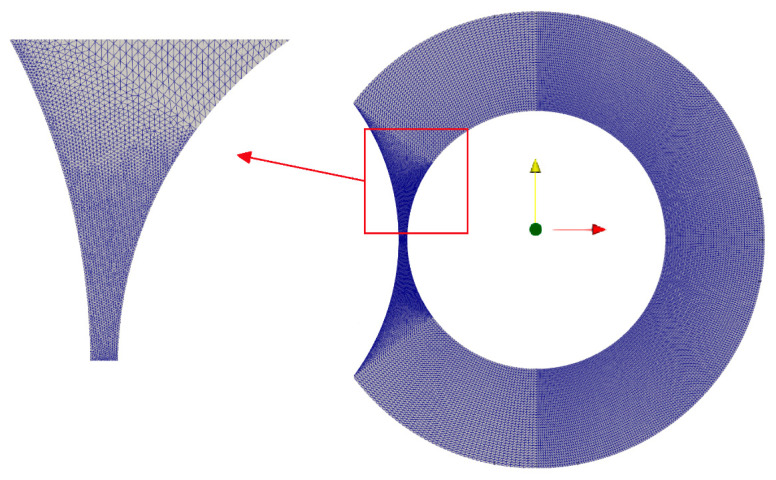
Numerical mesh employed for the simulations with structured and unstructured local regions, including a magnified view near the calender gap.

For the discretization of the computational domain, we use a combination of regular and unstructured meshes consisting of triangular volumes. All meshes are constructed with the GMSH software [35]. The unstructured grid is used to discretize the local curved domain in the calendering gap. A sample of the mesh is shown in Figure 7. The number of the volumes used is approximately *M* = 3 × 10^4^. This mesh is chosen after a mesh-independence study. We have constructed a coarse mesh of *M_c_* = 10^4^ volumes and a dense mesh with *M*_d_ = 9 × 10^4^ elements. Subsequently, we compared the values of the maximum velocity and the pressure at the center of the minimum calender gap. We found that these values changed by less than 0.1% from mesh *M* to *M*_c_. For boundary conditions, we assumed the no-slip condition at the surface of the barrel and the surface of the screws. The governing equations are solved iteratively using the SIMPLE [36] pressure-velocity correction loop [37]. For the solution of the system of the linear equations, a Preconditioned Conjugate Gradient (PCG) with Geometric agglomerated Algebraic MultiGrid (GAMG) preconditioner for the pressure and the velocity is used. The tolerance for both the pressure and the velocity is set to 10^−7^.

**Figure 8:**
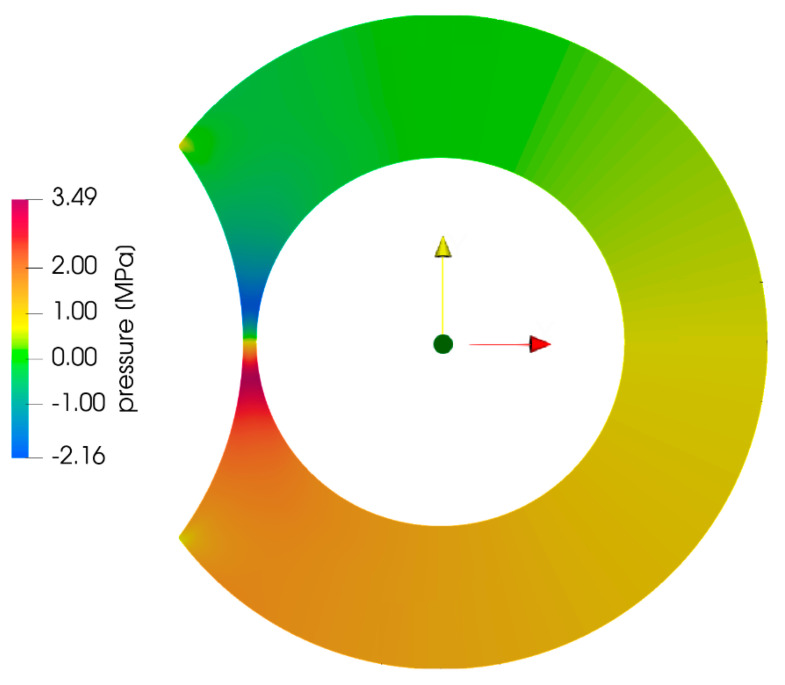
Pressure distribution obtained from numerical simulation for resin A (zero base pressure).

Figure 8 shows the numerically determined pressure field for resin A. As both screws rotate, the material is dragged towards the nip region, where the pressure rises to a maximum value just upstream of the minimum gap. Right after the minimum gap, the numerical simulation shows that the pressure drops to rather large negative values, which are meaningless [38]. Negative pressures have appeared in a number of published simulations [39,40,41,42] of flow in polymer processing machinery. The authors either do not discuss the issue or dismiss it as unimportant, insisting that the pressure gradient is the only thing that matters. However, in Figure 8, the negative pressures, in the divergent section, cancel out the positive pressures generated in the convergent section, resulting in zero separating force, which is not supported by experimental evidence.

**Figure 9:**
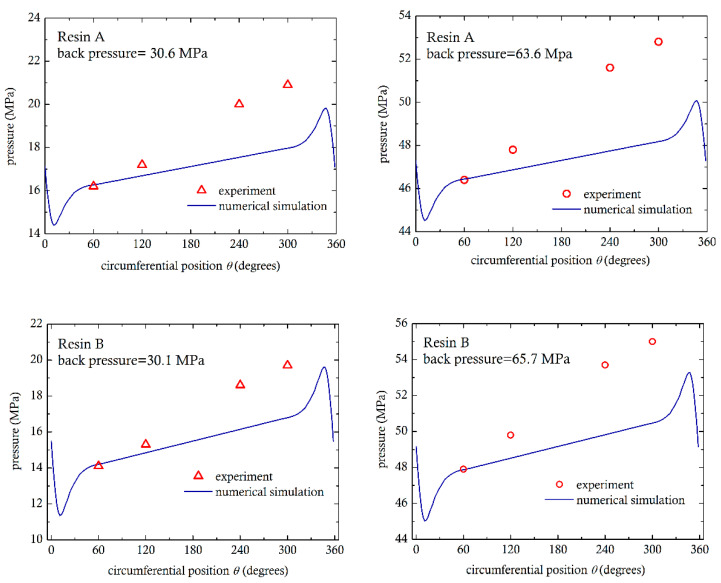
Comparison of experimental measurements of pressure with numerical simulations. Top row is for resin A and bottom row for resin B.

In Figure 9, experimental measurements for four representative cases (two for resin A and the other two for resin B) are compared to the simulations. The pressure as a function of position was obtained by setting the pressure level in the numerical simulations to the measured pressure at location P5.1, along the radius at 60° from the minimum gap. The numerical results, for all cases, predict a linear pressure rise, from 60° to 300°, qualitatively similar to the experiments. The differences are probably due the effect of the flights, which pass in front of the pressure sensors and generate their own local pressure rise. This effect could not be taken into consideration in the present two-dimensional simulations. Additionally, the numerical simulations predict pressure peaks in the convergent section and valleys in the divergent, which could not be measured experimentally.

**Figure 10:**
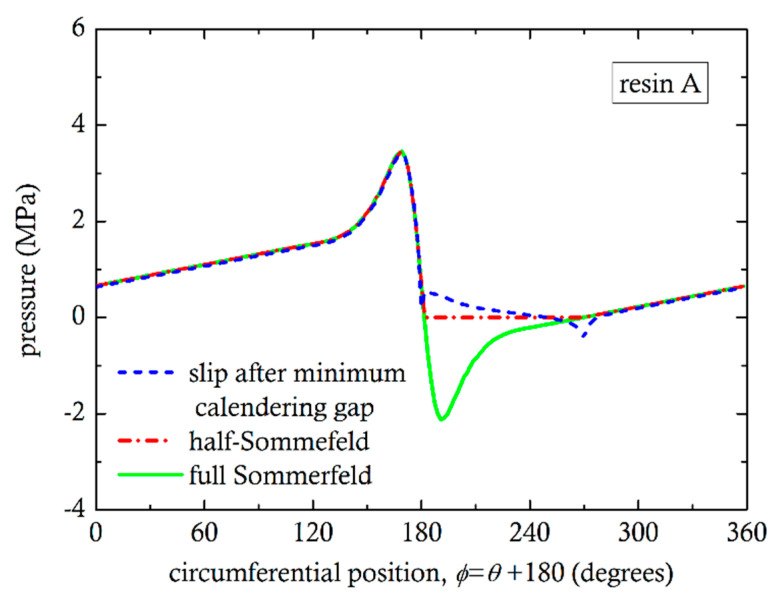
Effect of different conditions on the numerical pressure profile for resin A.

**Figure 11:**
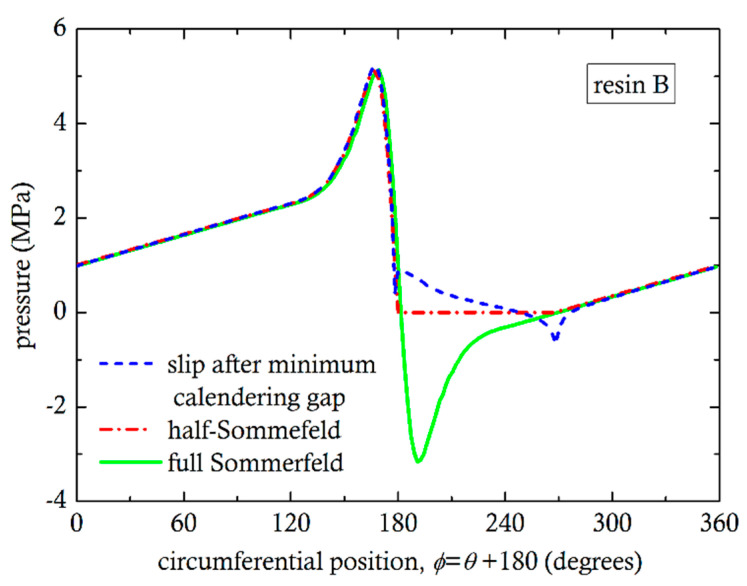
Effect of different conditions on the numerical pressure profile for resin B.

The problem of pressure peaks and valleys canceling each other has been studied extensively [20,43,44] in tribology, for the determination of load bearing capacity of journal bearings. The no-slip condition in the divergent region is responsible for the negative pressures and it is referred to as the Sommerfeld condition. Frequently, the so-called half-Sommerfeld condition has been used, by setting the pressure equal to zero where the Sommerfeld condition predicts negative pressure values [44]. Recent studies include cavitation models for the correct prediction of pressure in the divergent section. Cavitation in liquids occurs when the pressure becomes lower than the vapor pressure [21]. In molten polymer flows, there has not been much evidence of cavitation phenomena, except by Son and Migler [45] for polyethylene in connection with extrusion instabilities. It was concluded that cavitation is initiated very close to the die exit due to “reduced pressure and extensional stress”. In the calender gap, there are both reduced pressures and extensional flow. In the present case of a highly pressurized system, the strong extensional stresses are likely to produce cavitation phenomena in the divergent section. It is hypothesized that at the screw core and flight surfaces, slip is likely, due to cavitation. For this reason, simulations were also carried out assuming wall slip on the flight wall of Figure 1 and in the divergent section, on the screw root, from minimum gap up to 90°, but no slip on any section of the barrel. The pressure as a function of circumferential position for the three conditions (Sommerfeld, half-Sommerfeld, wall slip) are shown in Figure 10 and Figure 11. By integrating the pressures using Equations (4) and (5), we can easily obtain the angle of the resultant force from the vertical axis y. For Sommerfeld, it is very close to 0° for resin A and B, which means that there is no force in the x-direction. This result is totally against experimental evidence. For half-Sommerfeld, the angle is 18.38° and 18.4° and for the wall slip 23.93° and 24.87°, as shown in Table 4. These values are reasonably close to those obtained from the analysis of the experimental results (30°).

## 5. Discussion

The determination of the resultant forces on the screw core has been accomplished by integrating the local pressure forces around the circumference. The experimental measurements of pressure were made at 60°, 120°, 240° and 300° clockwise from the minimum gap and the values were fitted to straight lines. It was not possible to make measurements just upstream of the nip, where the pressure was expected to attain the highest value. It was decided to assume that the maximum pressure is reasonably approximated by extrapolations of the straight lines to 360°. The integrations gave resultant force angles of about 30° clockwise from the vertical. For the computer simulation, a two-dimensional flow analysis was carried out on a plane normal to the screw axis. As expected, negative pressures were obtained in the divergent section, which cancel the positive pressures in the convergent section. Using boundary conditions similar to those used in journal lubrication analyses, the negative pressures were removed and the subsequent integrations gave resultant force angles between 18° and 25°.

**Figure 12:**
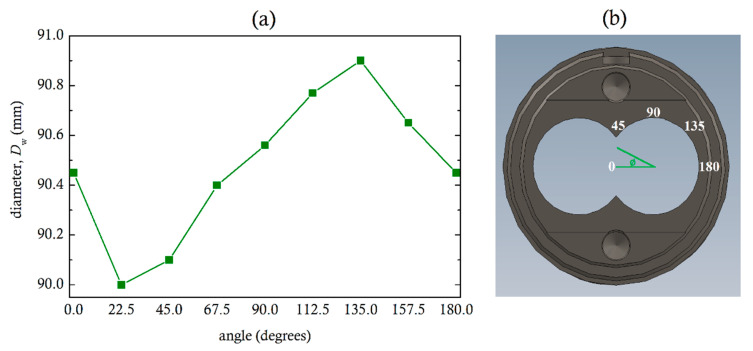
(**a**) Diameter measurements at different angles and (**b**) angle positions around the circumference of one cylinder.

In the industrial sector, it is frequently said that the screws move to directions popularly referred to as the ten o’clock and two o’clock positions, which correspond to 60° degrees from the vertical. Diameter measurements of the 90 mm extruder shown in Figure 2, were made with a Mitutoyo 511-703 Bore Gage, 100 mm from the exit. Figure 12 shows maximum diameter at the 135° angle position, which is 45° from the vertical.

In the present investigation (55 mm diameter extruder), the effect of gravity on the screw was not taken into account, because it is relatively small when compared to the separating forces. However, in large diameter extruders, a screw might be four meters long having a weight of a couple of tons. Such screws will be subjected to bending like cantilevered beams. The resultant force angle of calender separating force and gravity will be somewhat larger than those calculated in the present investigation.

## Figures and Tables

**Table 1:** The formulations of resin A and resin B.

Material	Resin A (phr)	Resin B (phr)
PVC, K value: 67	100	100
Calcium carbonate (CaCO_3_)	20	9
Stabilizer one-pack (Ca/Zn)	4	4.15
Titanium dioxide (TiO_2_)	3.7	5
Impact modifier	-	5.5
Solid density	1474 kg/m^3^	1448 kg/m^3^
Melt density	1327 kg/m^3^	1303 kg/m^3^

**Table 2:** Forces acting on a screw per meter of length and resultant force angles for resin A.

Experiment Case	Back Pressure (MPa)	*F_x_* (N)	*F_y_* (N)	Resultant Force Angle
L20-300	30.6	54,468	93,334	30.27°
L20-400	40.1	56,520	97,319	30.15°
L20-500	50.6	59,326	101,815	30.23°
L20-600	63.6	74,020	127,098	30.21°

**Table 3:** Forces acting on a screw per meter of length and resultant force angles for resin B.

Experiment Case	Back Pressure (MPa)	*F_x_* (N)	*F_y_* (N)	Resultant Force Angle
T20-300	30.1	64,739	110,951	30.26°
T20-400	40.4	67,216	115,714	30.16°
T20-500	49.9	70,261	121,134	30.11°
T20-600	60.5	77,061	132,532	30.18°
T50-650	65.7	79,761	139,394	29.79°

**Table 4:** Numerically determined forces acting on a screw per meter of length and resultant force angles for resin A and B under different conditions.

Condition	*F_x_* (N)	*F_y_* (N)	Resultant Force Angle
**Resin A**			
Half-Sommerfeld	13,086	39,387	18.38°
Wall slip	16,987	38,273	23.93°
**Resin B**			
Half-Sommerfeld	19,545	58,783	18.4°
Wall slip	26,954	58,409	24.87°

## References

[1] White J.L., Potente H. (2003). Screw Extrusion. Science and Technology.

[2] White J.L., Kim E.K. (2010). Twin Screw Extrusion. Technology and Principles.

[3] Tadmor Z., Gogos C.G. (2006). Principles of Polymer Processing.

[4] Janssen L.P.B.M. (1978). Twin Screw Extrusion.

[5] Martelli F. (1983). Twin–Screw Extruders: A Basic Understanding.

[6] Martin C., Vlachopoulos J., Wagner J.R. (2001). Twin-Screw Extruders. The SPE Guide on Extrusion Technology and Troubleshooting.

[7] Vlachopoulos J., Polychronopoulos N.D. (2019). Understanding Rheology and Technology of Polymer Extrusion.

[8] Midleman S. (1977). Fundamentals of Polymer Processing.

[9] Vlachopoulos J., Hrymak A.N. (1980). Calendering of PVC: Theory and Experiments. Polym. Eng. Sci..

[10] Mitsoulis E., Vlachopoulos J., Mirza F.A. (1985). Calendering analysis without the lubrication approximation. Polym. Eng. Sci..

[11] Polychronopoulos N.D., Vlachopoulos J., Jafar Mazumder M., Sheardown H., Al-Ahmed A. (2019). Polymer processing and rheology. Functional Polymers. Polymers and Polymeric Composites: A Reference Series.

[12] Polychronopoulos N.D., Sarris I.E., Papathanasiou T.D. (2014). 3D features in the calendering of thermoplastics: A computational investigation. Polym. Eng. Sci..

[13] Thiele W.C., Todd D.B. (1998). Counterrotating intermeshing twin-screw extruders. Plastics Compounding.

[14] Gomez I.L. (1984). Engineering with Rigid PVC: Processability and Applications.

[15] Rauwendaal C. (2014). Polymer Extrusion.

[16] Mennig G. (1995). Wear in Plastics Processing.

[17] Schneider H.-P., Liebhold J. (2013). Wear protection on twin screws. Kunstst. Int..

[18] Demirci A., Teke I., Goger A., Canba E., Vlachopoulos J. (2019). Gelation of poly (vinyl chloride) inside a single screw extruder and its effect on product properties. J. Vinyl Add. Tech..

[19] Leal L.G. (1992). Laminar Flow and Convective Transport. Processes.

[20] Khonsari M., Booser E.R. (2008). Applied Tribology: Bearing Design and Lubrication.

[21] Vlachopoulos J. (2016). Fundamentals of Fluid Mechanics.

[22] Wilczynski K., Nastaj A., Lewandowski A., Wilczynski K.J., Buziak K. (2019). Fundamentals of global modeling for polymer extrusion. Polymers.

[23] Janssen L.P.B.M., Mulders L.H.R.M., Smith J.M. (1975). A model from the output of the pump zone of the double screw processor or extruder. Plastics Polym..

[24] Speur J.A., Mavridis H., Vlachopoulos J., Janssen L.P.B.M. (1987). Flow patterns in the calender gap of a counterrotating twin screw extruder. Adv. Polym. Technol..

[25] White J.L., Adewale A. (1993). A unified view of modeling flow in counter-rotating twin screw extruders. Int. Polym. Proc..

[26] Li T., Manas-Zloczower I. (1994). Flow field analysis of an intermeshing counter-rotating twin screw extruder. Polym. Eng. Sci..

[27] Kajiwara T., Nagashima Y., Nakano Y., Funatsu K. (1996). Numerical study of twin-screw extruders by three-dimensional flow analysis-development of analysis technique and evaluation of mixing performance for full flight screws. Polym. Eng. Sci..

[28] Hong M.-H., White J.L. (1998). Fluid mechanics of intermeshing counter-rotating twin screw extruders. Int. Polym. Proc..

[29] Hong M.H., White J.L. (1999). Simulation of flow in an intermeshing modular counter-rotating twin screw extruder: Non-newtonian and non-isothermal behavior. Int. Polym. Proc..

[30] Schneider H.-P. (2005). The historical development of the counter-rotating twin-screw extruder. Kunstst. Plast. Eur..

[31] Wilczynski K., Lewandowski A. (2014). Study on the polymer melt flow in a closely intermeshing counter-rotating twin screw extruder. Int. Polym. Proc..

[32] Weller H., OpenFOAM CFD Direct Home Page. http://cfd.direct/about/henry-weller/.

[33] Polychronopoulos N.D., Papathanasiou T.D. (2015). A study on the effect of drawing on extrudate swell in film casting. Appl. Rheol..

[34] Polychronopoulos N.D., Vlachopoulos J. (2018). Computer flow simulation of moffatt eddies in single screw extrusion. Int. Polym. Proc..

[35] Geuzaine C., Remacle J.-F. (2009). Gmsh: A 3-D finite element mesh generator with built-in pre- and post-processing facilities. Int. J. Numer. Meth. Eng..

[36] Patankar S.V., Spalding D.B. (1972). A calculation procedure for heat, mass and momentum transfer in three-dimensional parabolic flows. Int. J. Heat Mass Transfer..

[37] Ternik P. (2010). New contributions on laminar flow of inelastic non-newtonian fluid in the two dimensional symmetric expansion: Creeping and slowly moving flow conditions. J. Nonnewtonian Fluid Mech..

[38] Goger A., Vlachopoulos J. (2014). Negative Pressures in modelling rotating polymer processing machinery are meaningless, but they are telling something. Int. Polym. Process..

[39] Gupta M., Rohatgi V., Kuehn R. (2009). Estimation of temperature increase in a ZSK-90 co-rotating twin-screw extruder using mesh partitioning technique. SPE ANTEC Tech. Pap..

[40] Ilinca F., Hetu J.-F. (2012). Three-dimensional numerical study of the mixing behaviour of twin-screw elements. Int. Polym. Process..

[41] Ishikawa T., Kihara S.-I., Funatsu K. (2000). 3-D numerical simulations of nonisothermal flow in co-rotating twin screw extruders. Polym. Eng. Sci..

[42] Radl S., Tritthart T., Khinast J.G. (2010). A novel design for hot-meltextrusion pelletizers. Chem. Eng. Sci..

[43] Dowson D., Taylor C.M. (1979). Cavitation in bearings. Ann. Rev. Fluid Mech..

[44] Concli F. (2016). Pressure distribution in small hydrodynamic journal bearings considering cavitation: A numerical approach based on the open-source CFD code OpenFOAM. Lubr. Sci..

[45] Son Y., Migler K.B. (2002). Cavitation of polyethylene during extrusion processing instabilities. J. Polym. Sci. B Polym. Phys..

